# The impact of thrombus surface morphology of the basilar artery on the successful rate of mechanical thrombectomy

**DOI:** 10.3389/fneur.2023.1280317

**Published:** 2023-11-21

**Authors:** Xinzhao Jiang, Shufeng Yu, David Wang, YI Dong, Peng Wang

**Affiliations:** ^1^Center for Rehabilitation Medicine, Department of Neurology, Zhejiang Provincial People’s Hospital (Affiliated People’s Hospital), Hangzhou Medical College, Hangzhou, Zhejiang, China; ^2^Center for Rehabilitation Medicine, Department of Radiology, Zhejiang Provincial People’s Hospital (Affiliated People’s Hospital), Hangzhou Medical College, Hangzhou, Zhejiang, China; ^3^Department of Neurology, Barrow Neurological Institute (BNI), Phoenix, AZ, United States; ^4^Department of Neurology, Huashan Hospital, Fudan University, Shanghai, Shanghai Municipality, China

**Keywords:** thrombus morphology, basilar artery occlusion, mechanical thrombectomy, acute stroke, contact aspiration, stent retriever

## Abstract

**Objective:**

The present study aimed to investigate the impact of angiographic thrombus surface morphology on the angiographic and clinical outcomes of basilar artery occlusion (BAO).

**Methods:**

This retrospective study included 141 patients with acute BAO who underwent mechanical thrombectomy (MT). We categorized thrombus surface phenotypes as either regular (smooth and straight, either convex or concave) or irregular. Patients with BAO were grouped based on the presence of a regular or irregular phenotype, and we compared their angiographic and clinical outcomes.

**Results:**

In total, 52.5% (74/141) of acute BAO patients exhibited a regular thrombus morphology. These patients had a higher rate of first-pass effect (28.4% vs. 4.5%, *p* = 0.0002) and fewer retrieval attempts (2 vs. 2; *p* = 0.0198) compared to those with irregular morphology. Among patients treated with contact aspiration (CA), the regular thrombus morphology showed a higher first-pass success rate (45.7% vs. 12.8%; *p* = 0.0017), a shorter procedural duration (46 vs. 50 min; *p* = 0.0159), and fewer retrieval attempts (1 vs. 2; *p* = 0.0338) compared to stent retriever (SR) thrombectomy. Both the regular thrombus morphology (OR 7.72, 95% CI 2.02–29.52; *p* = 0.003) and using CA as the first-line treatment (OR 3.37, 95% CI 1.12–10.13; *p* = 0.031) independently predicted first-pass success.

**Conclusion:**

For BAO patients treated with CA as the primary strategy, the presence of a regular thrombus surface might predict higher first-pass success and shorter procedural duration. A diligent assessment of thrombus morphology within the MT workflow could improve the feasibility of procedural techniques.

## Introduction

Treatment outcomes for patients with basilar artery occlusion (BAO) have been less than optimal, despite recent advancements in mechanical thrombectomy (MT) techniques (1–3). When compared to anterior circulation cases, some BAO patients continue to exhibit poorer clinical results (4). This discrepancy may be attributed to distinct anatomical variations in the posterior circulation, including vessel caliber, vessel tortuosity, perforators, and collateral patterns (5, 6). Additionally, intracranial atherosclerotic stenosis (ICAS) is a more frequent cause of posterior circulation artery occlusion compared to the anterior circulation (6). Nevertheless, achieving timely and successful recanalization remains crucial for optimal outcomes in BAO patients (7, 8).

Recent attention has focused on predictive radiological features, such as the meniscus sign, cut-off sign, and tapered sign, which may enhance the likelihood of rapid and successful recanalization (9). Prior research has suggested that when the initial digital subtraction angiography (DSA) reveals a regular thrombus surface in anterior circulation large vessel occlusion (LVO), it is associated with higher recanalization rates, particularly when using contact aspiration (CA) as the first-line thrombectomy device (9–13). However, it remains uncertain whether the impact of this thrombus surface characteristic also applies to BAO patients undergoing MT. Therefore, our study aims to validate these findings by assessing the influence of a regular thrombus surface on the success rate and the selection of the first-line device in BAO patients treated with MT.

## Methods

### Patient and study design

**Figure 1:**
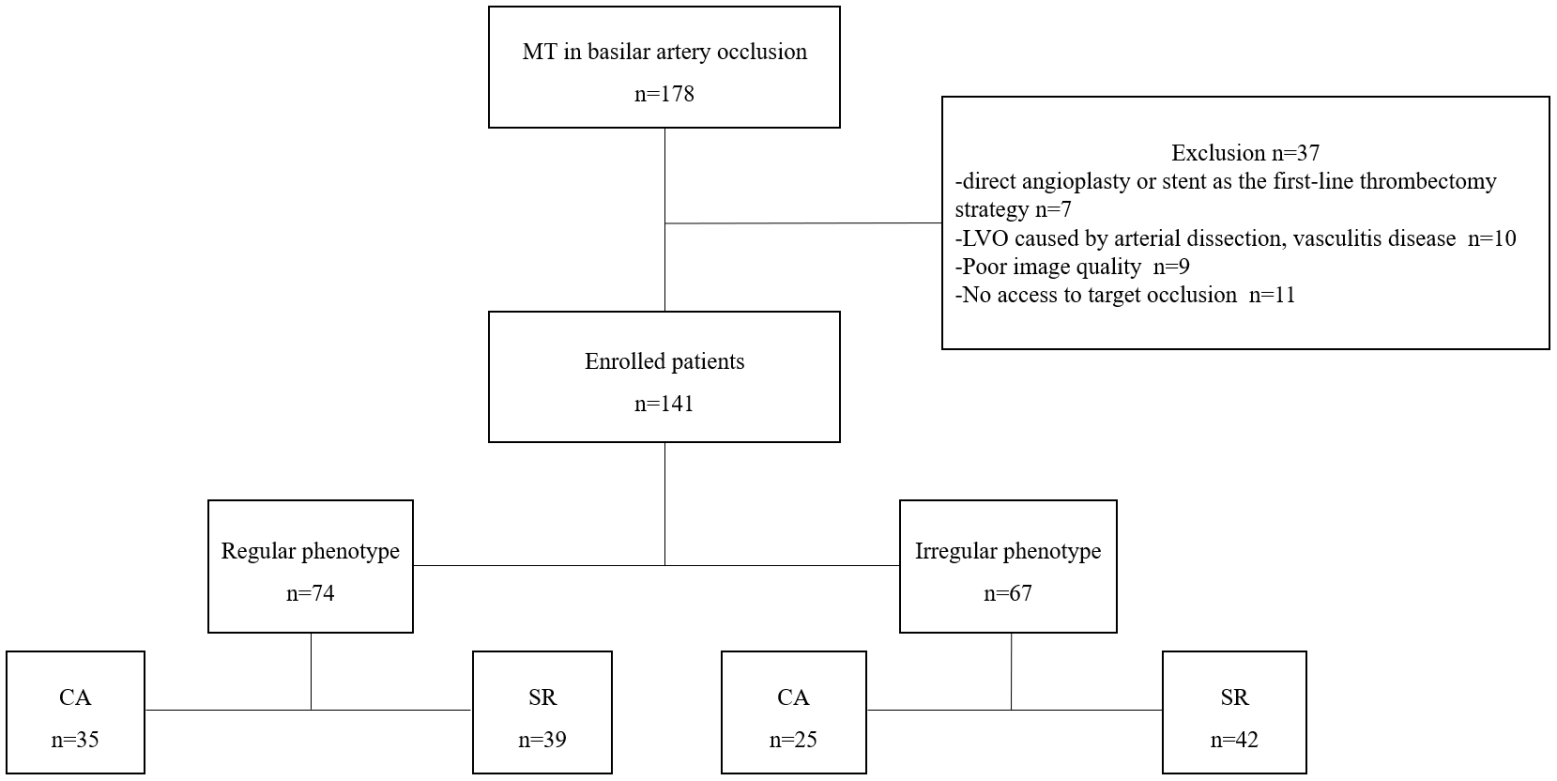
Flow chart of patient selection. MT, mechanical thrombectomy; LVO, large vessel occlusion; CA, contact aspiration; SR, stent retriever.

The data were obtained from the prospective registry of our stroke database. All consecutive patients with BAO treated with MT between January 2018 and June 2022 were enrolled if they met the following inclusion criteria: (1) acute symptomatic patients with BAO identified on computed tomographic (CT) or magnetic resonance (MR) angiography; (2) patients with a pre-stroke modified Rankin Scale (mRS) 0–2 (14); (3) patients treated with stent retriever or contact aspiration as the primary treatment; and (4) time from onset of symptoms to groin puncture of ≤12 h. They were excluded if the following was true: (1) SR or CA was not the first-line thrombectomy strategy; (2) the target vascular occlusion could not be reached with thrombectomy devices; (3) LVO was caused by arterial dissection, vasculitis, or moyamoya disease; and (4) angiography images were of poor quality. The patient selection process is shown in Figure 1.

The following data were also collected: patient’s demographics, comorbidities, vascular risk factors, symptom onset, and severity of stroke: National Institutes of Health Stroke Scale (NIHSS) and Posterior Circulation Alberta Stroke Program Early CT Score (PC-ASPECTS), treatment with intravenous thrombolysis (IVT), the degree of collateral circulation(good or poor) (15), endovascular devices, total attempts, times, and recanalization results [modified treatment in cerebral ischemia (mTICI), clinical outcomes such as mRS after 90 days, and symptomatic intracerebral hemorrhage (ICH)]. This retrospective study was approved by the local ethics committee of Zhejiang Provincial People’s Hospital. (NO.KY2017019). The need for written informed consent was waived given the retrospective nature of this study.

### Assessment of thrombus surface phenotype

**Figure 2:**
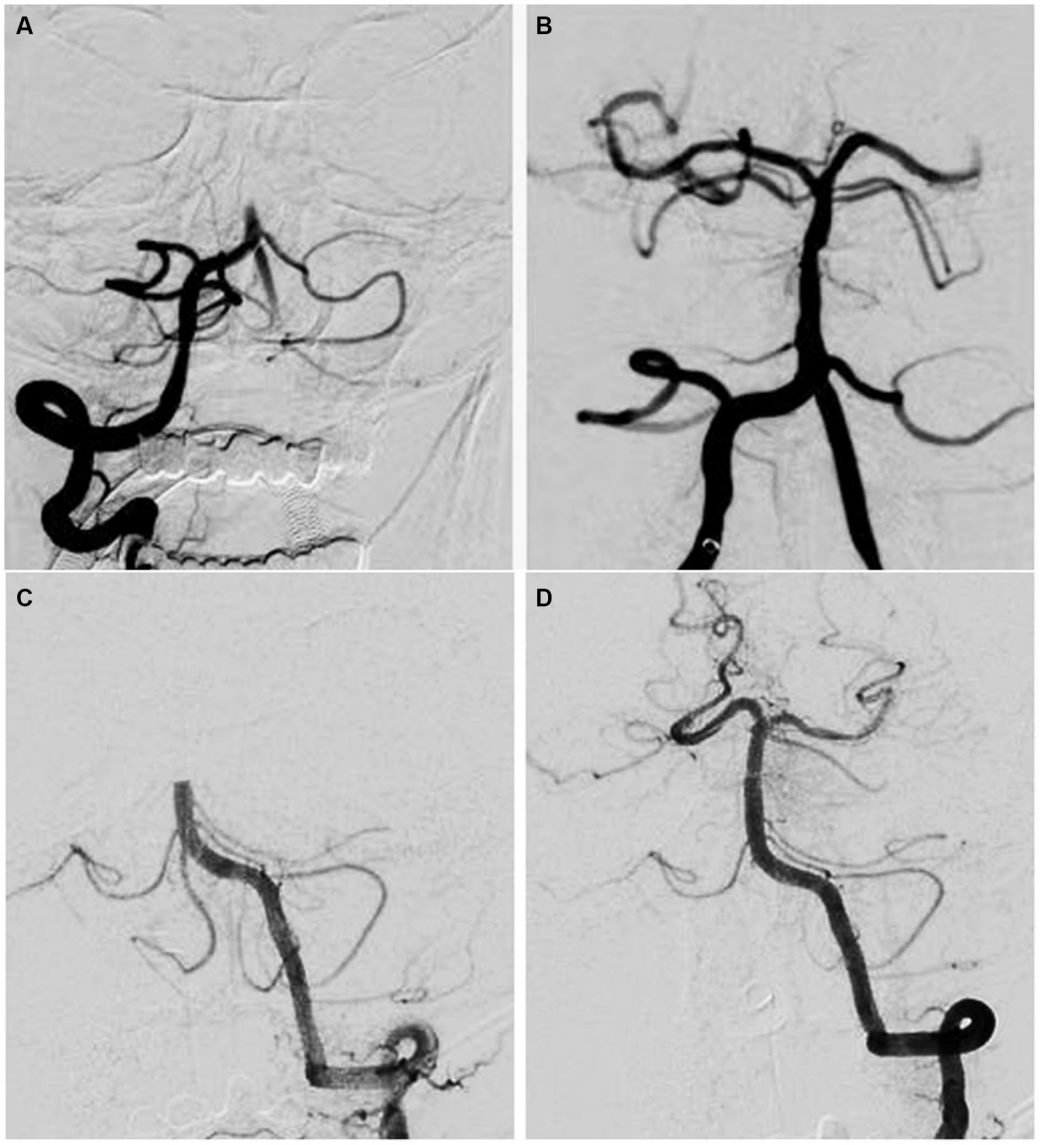
Pre-treatment digital subtraction angiography images of basilar artery occlusion with different thrombus surface phenotypes of the occlusion site. **(A,B)** Pre-treatment digital subtraction angiogram shows the irregular phenotype of an occlusion at the proximal segment of the basilar artery. After multiple attempts of mechanical thrombectomy, complete recanalization of the basilar artery with residual stenosis is seen. **(C,D)** Pre-treatment digital subtraction angiogram shows the regular phenotype of an occlusion at the middle segment of the basilar artery. After a single attempt of contact aspiration, complete recanalization of the basilar artery without residual stenosis is seen.

The thrombus surface phenotype of the target vessel occlusion was classified as “regular” if the profile of the occlusion was smooth and straight with a linear interruption of flow in the artery that appeared to be abruptly cut off; an irregular pattern was classified as any type of obvious change in the linearity of the occlusion, such as concave or convex shapes or the presence of multiple contrast filling defects on the profile of the occlusion (Figure 2). Two experienced neuroradiologists (W.P, D.Y.) with >10 years’ experience in cerebrovascular imaging evaluated the thrombus surface phenotypes. During imaging analysis, the reviewers were blinded to the device usage, angiographic data, and clinical outcome. Any controversy was resolved by the consensus of two reviewers.

### Outcome measures

Recanalization status was classified according to the modified Thrombolysis in Cerebral Infarction (TICI) scale. The angiographic outcomes of the present study included the first pass effect (FPE), successful recanalization (mTICI 2b-3) after procedure, number of passes, rescues treatment, and procedure time. The first pass effect was defined as achieving complete recanalization (mTICI 3) with a single thrombectomy device and without any rescue treatment (16, 17). The procedure time was defined as the time from puncture to final recanalization in those with successful recanalization or time of the last angiographic series in those with unsuccessful recanalization (18). Clinical outcomes were measured with mRS after 90 days and post-procedure ICH. Favorable clinical outcome was defined as a 90-day mRS score of 0–2.

### Statistical analysis

The baseline characteristics, angiographic outcome, clinical outcomes, and complications were compared hierarchically based on ‘regular’ or ‘irregular’ thrombus surface phenotype. The results were reported as frequency, mean ± standard deviation (SD), or median (interquartile range, IQR). The overall sample used Pearson’s χ2-test or Fisher’s exact test for categorical variables. In contrast, the Student’s t-test or Mann–Whitney U test (for non-Gaussian distributions) is used for continuous variables comparison. Univariate and Multivariable logistic regression were performed to evaluate the independent variables of first-pass effect. The inter-rater reliability of occlusion type rating was calculated using Cohen’s kappa coefficient (k) with its 95% confidence interval (CI). Kappa values of 0–0.2 indicate slight, 0.21–0.4 fair, 0.41–0.6 moderate, 0.61–0.8 substantial, and 0.81–1.00 almost perfect agreement, respectively^17^. A *p* < 0.05 was considered statistically significant. SPSS Statistics (Version 25) and R software (version 3.5.3) were used for all statistical analyses.

## Results

During the study period, 178 consecutive patients underwent MT due to BAO. Among them, 37 patients were excluded due to the following reasons: direct angioplasty or stent as the first-line treatment (*n* = 7), occluded BA due to dissection or vasculitis (*n* = 10), poor image quality (*n* = 9), and no access to target occlusion (*n* = 11). Finally, 141 patients were included in the present study. Baseline characteristics including comorbidities and vascular risk factors are shown in Table 1. The median age was 65 (IQR 37–87), and 68.8% (97/141) were male. The baseline NIHSS and ASPECT on admission were 15 (IQR 8–27) and 8 (IQR 7–9), respectively. IVT was administered to 61 patients (43.3%) before EVT. The average time from symptom onset to groin puncture was 290 ± 74 min. A regular thrombus surface was diagnosed in 52.5% (74/141). For the regular or irregular thrombus surface, we found an excellent inter-reader and intra-reader agreement (κ values, 0.87, 95% CI 0.74 to 1.0, and 0.92, 95% CI 0.87 to 0.95, respectively). The baseline characteristics between regular and irregular groups did not differ significantly.

**Table 1:** Comparison of baseline characteristics, treatment, and outcomes of basilar artery occlusion patients according to regular/irregular phenotype.

	Total (*n* = 141)	Regular (*n* = 74)	Irregular (*n* = 67)	*p* value
Baseline characteristics				
Age (years) [median (IQR)]	65 (37–87)	65 (37–84)	63 (39–87)	0.1474
Sex (male)	97 (68.8)	49	48	0.4874
Admission NIHSS [median (IQR)]	15 (8–27)	15 (8–27)	15 (9–27)	0.9835
ASPECT [median (IQR)]	8 (7–9)	8 (7–9)	8 (7–9)	0.9125
Hypertension	93 (65.9)	44	49	0.087
Diabetes	49 (34.8)	23	26	0.3361
Dyslipidemia	44 (31.2)	23	21	0.9732
Atrial fibrillation	62 (43.9)	33	29	0.8755
Coronary artery disease	45 (31.9)	21	24	0.3438
Smoking	70 (49.6)	35	35	0.5578
Intravenous tPA	61 (43.3)	34	27	0.4991
Onset to puncture time (min) [mean (SD)]	290 (74)	284 (77)	297 (70)	0.5088
*Occlusion site*				
Proximal	42 (29.8)	22	20	0.9875
Middle	46 (32.6)	25	21	0.7576
Distal	53 (37.6)	27	26	0.7764
First-line device				0.2311
CA	60 (42.6)	35	25	
SR	81 (57.4)	39	42	
Collateral circulation				0.7910
Good	69(48.9)	37(50)	32(47.8)	
Poor	72(51.1)	37(50)	35(52.2)	
*Angiographic outcomes*				
First pass effect *	24 (17.0)	21 (28.4)	3 (4.5)	0.0002
End of procedure mTICI 2b-3	126 (89.4)	69 (93.2)	57 (85.1)	0.1162
Number of passes [median (IQR)] *	2 (1–4)	2 (1–3)	2 (1–4)	0.0198
Procedure time (min) [mean (SD)]	48 (7)	48 (6)	49 (7)	0.2361
Rescue treatment	31 (21.9)	14 (18.9)	17 (25.4)	0.3554
*Clinical outcomes*				
90-day mRS 0–2	62 (43.9)	34	28	0.6196
90-day mRS 6	19 (13.5)	10	9	0.9888
Symptomatic ICH	9 (6.4)	5	4	0.9

Of all patients, 17.0% (*n* = 24/141) presented with the first pass effect. Patients with regular thrombus surface had a higher rate of first pass effect compared to those with irregular thrombus surface (28.4% vs. 4.5%; *p* = 0.0002). There were no significant differences in this cohort with either regular or irregular thrombus surface morphology in their rate of successful recanalization (mTICI 2b-3) after the procedure (93.2% vs. 85.1%; *p* = 0.1162), the procedure time (48 vs. 49 min; *p* = 0.2361), and number of rescue treatment (18.9% vs. 25.4%; *p* = 0.3554). The mean procedure time was 48 ± 7 min, and the median number of passes was 2 (IQR 1–4). Patients with a regular thrombus surface had a significantly lower median number of passe than irregular thrombus surface [2 (IQR 1–3) vs. 2 (IQR 1–4), *p* = 0.0198]. Rescue treatment was reported in 21.9% (n = 31) of all patients (Table 1).

In the group with regular thrombus surface, patients treated with CA had a higher rate of FPE (45.7% vs. 12.8%, *p* = 0.0017), lower number of passes (1 vs. 2, *p* = 0.0388) and shorter procedure time (46 vs. 50, *p* = 0.0159). In the group with irregular thrombus surface, patients treated with SR had a lower rate of rescue treatment (16.7% vs. 40.0%; *p* = 0.0338). However, there was no significant difference in successful recanalization, favorable clinical outcome (90-day mRS 6), symptomatic ICH after the procedure, and occlusion site between the CA and SR groups in patients with a regular/irregular thrombus surface (Table 2).

**Table 2:** Treatment and clinical outcomes of the CA and SR groups according to regular/irregular phenotype.

	Regular phenotype		*p* value	Irregular phenotype		*p* value
	CA (*n* = 35)	SR (*n* = 39)		CA (*n* = 25)	SR (*n* = 42)	
First pass effect*	16 (45.7)	5 (12.8)	0.0017*	1 (4.0)	2 (4.8)	0.9
End of procedure mTICI 2b-3	32 (91.4)	37 (94.9)	0.6624	21 (84.0)	36 (85.7)	0.9
Number of passes [median (IQR)]^†^	1 (1–3)	2 (1–3)	0.0388^†^	2 (1–4)	2 (1–4)	0.972
Procedure time [mean (SD)]^‡^	46 (5)	50 (6)	0.0159^‡^	50 (9)	50 (7)	0.9019
Rescue treatment ^	6 (17.1)	8 (20.5)	0.7117	10 (40.0)	7 (16.7)	0.0338^
90-day mRS 0–2	17 (48.6)	17 (43.6)	0.6677	10 (40.0)	18 (42.9)	0.8186
90-day mRS 6	5 (14.3)	5 (12.8)	0.9	4 (16.0)	5 (11.9)	0.7178
Symptomatic ICH	2 (5.7)	3 (7.7)	0.9	1 (4.0)	3 (7.1)	0.9
Occlusion site						
Proximal	10 (28.6)	12 (30.8)	0.8364	9 (36.0)	11 (26.2)	0.3961
Middle	14(40.0)	11 (28.2)	0.2841	7 (28.0)	14 (33.3)	0.6490
Distal	11(31.4)	16 (41.0)	0.3919	9 (36.0)	17 (40.5)	0.7161

Symbols (*, ^†^, ^‡^, ^) indicate group comparisons, which express that there is significant difference between CA and SR groups.

Multivariate logistic regression analysis showed that the regular thrombus surface (OR 7.72, 95% CI 2.02–29.52; *p* = 0.003) and the first-line CA thrombectomy (OR 3.37, 95% CI 1.12–10.13; *p* = 0.031) were independent predictors of PFE when adjusting for sex (male), atrial fibrillation, and procedure time (Table 3).

**Table 3:** Univariate and Multivariable logistic regression analysis for first-pass effect.

	First-pass effect			
Variables	Univariate analysis		Multivariate analysis	
	OR (95% CI)	*p* value	OR (95% CI)	*p* value
Age	1.022 (0.981–1.065)	0.301		
Sex (male)	0.182 (0.041–0.813)	0.026	0.200 (0.040–1.003)	0.05
Admission NIHSS	0.937 (0.840–1.044)	0.239		
PC-ASPECT	0.858 (0.486–1.515)	0.598		
Hypertension	0.673 (0.274–1.655)	0.389		
Diabetes	0.574 (0.212–1.555)	0.275		
Dyslipidemia	1.673 (0.678–4.128)	0.264		
Atrial fibrillation	3.087 (1.223–7.794)	0.017	2.714 (0.902–8.166)	0.076
Coronary artery disease	0.642 (0.236–1.744)	0.384		
Smoking	0.634 (0.260–1.541)	0.314		
Intravenous tPA	0.750 (0.304–1.851)	0.532		
Procedure time	1.004 (0.997–1.010)	0.249		
Regular phenotype	8.453 (2.390–29.898)	0.001	7.724 (2.021–29.518)	0.003
First-line CA	4.179 (1.605–10.883)	0.003	3.366 (1.118–10.133)	0.031
Procedure time	0.880 (0.810–0.956)	0.002	0.902 (0.813–1.001)	0.052
Distal occlusion	1.854 (0.764–4.495)	0.172		

CA, contact aspiration; tPA, tissue-type plasminogen activator.

## Discussion

This study underscores the significant impact of angiographic thrombus surface morphology on the treatment outcomes of acute BAO patients undergoing MT. The presence of a regular thrombus surface is closely associated with several positive treatment outcomes, including a higher first-pass success rate, a reduced total number of passes, and shorter procedure times. BAO patients with regular thrombus surfaces, particularly those treated with CA as the primary thrombectomy method, demonstrate superior first-pass success rates, shorter procedural durations, and fewer passes compared to those treated with stent retriever SR thrombectomy. It is important to note that the regular thrombus surface and the choice of CA as the first-line thrombectomy are independent predictors of first-pass success in BAO cases.

In our study, we defined a regular thrombus surface as one exhibiting a smooth and straight profile with a linear interruption of flow, giving the appearance of an abrupt cutoff in the artery. This definition aligns with similar classification methods described in previous studies. For instance, the clot meniscus sign can be considered a subset of the regular thrombus surface. Furthermore, a more detailed subtyping of thrombus phenotypes, such as the meniscus sign, may inadvertently exclude patients who could benefit from a phenotype-based device selection. Therefore, our regular/irregular thrombus surface definition is likely more suitable for clinical application.

In the context of anterior circulation large vessel occlusion (LVO), prior studies have explored the morphological analysis of occlusive lesions in relation to MT efficacy (10–13). Two studies have demonstrated higher success rates and complete first-pass recanalization (mTICI 2b-3 and mTICI 3) when employing first-line CA in patients with regular thrombus surfaces (10, 12). In contrast, one study has suggested that irregular M1-MCA occlusive thrombus surfaces treated with SR as the first-line thrombectomy device were associated with higher success rates and complete first-pass recanalization (11).

Regarding complete recanalization, recent research has indicated that, in BAO patients with the clot meniscus sign or a regular thrombus phenotype, first-line CA thrombectomy may be more effective than SR (5). Interestingly, among patients with irregular thrombus surfaces, we observed a significant difference in the rate of rescue treatments between the CA and SR groups, which is consistent with previous findings that noted a higher rate of rescue treatments in CA thrombectomy for M1-MCA occlusions compared to SR (11). However, in patients with irregular thrombus surfaces, we found no differences in recanalization rates, procedural times, or the total number of retrievals attempts between the CA and SR groups.

The existing literature on the association between thrombus surface phenotype and treatment outcomes in BAO patients is limited. Previous studies have indicated that meniscoid-like thrombus surfaces or the claw sign are associated with higher recanalization (18, 19). A recent study suggested that the regular thrombus surface may independently predict complete recanalization with first-line CA in BAO patients (20). However, these studies did not further investigate the impact of thrombus surface phenotypes on recanalization efficacy with different first-line thrombectomy devices (CA or SR). Our study validated the results of the previous study in a relatively larger cohort and further demonstrated the relationship between the success of the first pass using two different recanalization techniques and thrombus surface phenotypes in BAO patients.

Achieving early complete recanalization is a primary goal in BAO and is pivotal for better clinical outcomes (7, 8). In this regard, first-line CA thrombectomy may be associated with higher rates of complete recanalization compared to SR (8). Our findings, including the independent prediction of complete recanalization after the first pass by the regular thrombus surface and first-line CA thrombectomy in BAO patients, align with our initial hypothesis and prior research (20). Nevertheless, our study did not reveal a significant difference in favorable clinical outcomes between patients with regular and irregular thrombus surfaces regardless of the technique employed (CA or SR). This finding is consistent with previous studies that found no significant difference in clinical outcomes based on thrombus surface morphology in BAO patients. (20)The lack of significant differences in clinical outcomes may be attributed to the current sample size, which may not yet balance the multitude of factors influencing clinical outcomes.

The association between recanalization efficacy and the regular/irregular thrombus surfaces of large vessel occlusions remains an area of ongoing exploration. While most studies have reported no significant difference in the rate of complete first-pass recanalization between thrombus surfaces (10, 11), recent literature has highlighted a potential link between the rate of complete first-pass recanalization and angiographic thrombus surfaces of occlusive lesions and found that patients with an anterior circulation LVO from a thrombus of regular surface had a higher rate of complete first-pass recanalization than those with an irregular thrombus surface (46.3% vs. 20.7%; *p* = 0.0004). Similarly, our study showed that BAO patients with regular thrombus surfaces had a higher rate of complete first-pass recanalization than those with an irregular thrombus surface (28.4% vs. 4.5%; *p* = 0.0002).

Our results indicate that the use of CA is associated with higher rates of FPE in patients with BAO who exhibit the regular thrombus phenotype. Several potential mechanisms may explain the superiority of CA over stent retriever (SR) thrombectomy in BAO patients with a regular thrombus surface: (1) During CA, a large-bore aspiration/intermediate catheter can occupy a higher percentage of the cross-sectional area of the vessel lumen. This effective vessel lumen occupation may reduce the impact force on the thrombus, resulting in more efficient retrieval and a decreased risk of thrombus distal migration (5, 18). (2) Enhanced “Contact”: Successful recanalization in CA thrombectomy relies on effective “contact” between the aspiration catheter and the proximal surface of the thrombus (21). The regular thrombus surface substantially increases the contact area of the aspiration catheter with the thrombus, potentially improving the catheter’s efficiency and increasing the revascularization rate (3). Erythrocyte-rich thrombus was associated with cardioembolic etiology (22). Our statistics and previous study showed that patients with atrial fibrillation had a higher proportion of regular thrombus surface than irregular thrombus surface. The characteristics of the erythrocyte-rich thrombus had a higher clot burden and higher density (23). The physiological characteristics may be increasing the catheter’s efficiency and decreased risk of thrombus distal migration. However, further studies are needed for revealing the precise association of histological thrombus composition with different surface phenotypes.

### Limitations

Our study has several limitations. Firstly, it employs a retrospective design, which warrants a cautious interpretation of the results. Secondly, the choice of the first-line thrombectomy device was determined by the interventionist’s experience, introducing potential bias. Thirdly, our study primarily involved Chinese populations, who exhibit a higher incidence of intracranial atherosclerotic stenosis (ICAS), which may influence the outcomes of endovascular treatment. Lastly, we did not conduct a histologic examination of the retrieved clots, leaving the relationship between thrombus composition and angiographic morphology as a potential avenue for future research.

## Conclusion

For BAO patients treated with CA as the primary strategy, the presence of a regular thrombus surface might predict higher first-pass success and shorter procedural duration. Diligent assessment of thrombus morphology within the MT workflow could improve the feasibility of procedural techniques.

## Data availability statement

The raw data supporting the conclusions of this article will be made available by the authors, without undue reservation.

## Ethics statement

The studies involving humans were approved by the local Ethics Committee of Zhejiang Provincial People’s Hospital. (No. KY2017019). The studies were conducted in accordance with the local legislation and institutional requirements. Written informed consent for participation was not required from the participants or the participants’ legal guardians/next of kin in accordance with the national legislation and institutional requirements.

## Author contributions

XJ: Writing – original draft, Writing – review & editing. SY: Writing – review & editing. DW: Conceptualization, Writing – original draft. YD: Supervision, Writing – review & editing. PW: Funding acquisition, Resources, Writing – original draft.
